# Assessment of molybdenum application on soybean physiological characteristics in maize-soybean intercropping

**DOI:** 10.3389/fpls.2023.1240146

**Published:** 2023-09-29

**Authors:** Zameer Hussain Jamali, Shahzaib Ali, Muhammad Qasim, Chun Song, Muhammad Anwar, Junbo Du, Yu Wang

**Affiliations:** ^1^ College of Environmental Sciences, Sichuan Agricultural University, Chengdu, China; ^2^ Department of Agroecosystems, Faculty of Agriculture and Technology, University of South Bohemia Ceske Budejovice, Ceske Budejovice, Czechia; ^3^ College of Resources and Environment, Huazhong Agricultural University, Wuhan, China; ^4^ School of Tropical Agriculture and forestry, Hainan University, Haikou, China; ^5^ College of Agronomy, Sichuan Agricultural University, Chengdu, China; ^6^ College of Landscape Architecture, Sichuan Agricultural University, Chengdu, China

**Keywords:** molybdenum, soybean, intercropping, physiological, nutrient-use-efficiency

## Abstract

Soybean is a leguminous crop known for its efficient nitrogen utilization and ease of cultivation. However, its intercropping with maize may lead to severe reduction in its growth and yield due to shading effect of maize. This issue can be resolved by the appropriate application of essential plant nutrient such as molybdenum (Mo). Aim of this study was to assess the effect of Mo application on the morphological and physiological characteristics of soybean intercropped with maize. A two-year field experiment was conducted for this purpose, and Mo was applied in the form of sodium molybdate (Na_2_MoO_4_), and four different levels were maintained i.e., 0, 60, 120 and 180 g ha^-1^. Soybean exhibited varying responses to different levels of molybdenum (Mo) application. Notably, in both sole and intercropped cropping systems, the application of Mo at a rate of 120 g ha^-1^ demonstrated the highest level of promise compared to other application levels. However, most significant outcomes were pragmatic in soybean-maize intercropping, as application of Mo @ 120 g ha^-1^ significantly improved soybean growth and yield attributes, including leaf area index (LAI; 434 and 441%), total plant biomass (430 and 461%), transpiration rate (15 and 18%), stomatal conductance (9 and 11%), and yield (15 and 20%) during year 2020 and 2021 respectively, as compared to control treatment. Similarly, Mo @ 120 g ha^-1^ application resulted in highest total grain yield (626.0 and 725.3 kg ha^-1^) during 2020 and 2021 respectively, which exceeded the grain yields of other Mo levels under intercropping. Moreover, under Mo application level (120 g ha^-1^), grain NPK and Mo contents during years 2020 and 2021 were found to be 1.15, 0.22, 0.83 and 68.94 mg kg^-1^, and 1.27, 0.25, 0.90 and 72.18 mg kg^−1^ under intercropping system increased the value as compared to control treatment. Findings of current study highlighted the significance of Mo in enhancing soybean growth, yield, and nutrient uptake efficiency in maize-soybean intercropping systems.

## 1 Introduction

Worldwide, agriculture is currently facing numerous challenges, including growing populations, land use change, urban sprawl, industrial development and climate change, all of which contribute to food security issues (Kalugina, 2014; Hu et al., 2016). One of the leading global challenges is to conserve natural resources, agricultural productivity, and biodiversity while addressing food security (Brown, 2002; Jiren et al., 2021). Soybean (*Glycine max* L.), a vital leguminous crop, is valued for its high-quality oil and protein, encompassing essential amino acids crucial for human health (Hou et al., 2015; Zeeshan et al., 2023). In 2019, global soybean production exceeded 333 million tons (Faostat, 2019; Jahan et al., 2023). Intercropping has been identified as a potential solution to promote sustainable agricultural development by improving land utilization rates and ensuring higher and sustainable crop yields (Bedoussac et al., 2015; Iqbal et al., 2019). Intercropping involves growing two or more crop species together for their specific cropping periods. Recent studies have shown that cassava intercropping is prevalent in America and Africa (Zhang et al., 2015). Legume-cereal intercropping, particularly maize-bean intercropping, is a common practice in East and Southern Africa (Mucheru-Muna et al., 2010; Fischer et al., 2020). Intercropping is also widely practice in Asian countries like China, such as wheat-maize (Gao et al., 2014), maize-soybean (Liu et al., 2018), sunflower-soybean (de la Fuente et al., 2014), and maize-pea (Teng et al., 2016). Despite vast potential and applicability of intercropping, certain challenges may arise, including nutrient competition, imbalances, and resource acquisition between co-existing crops (Huss et al., 2022). Intercropping also enables the adjacent crops to ensure greater yields owing to complimentary utilization of limited resources (Wang et al., 2023).

One of the leading concern in maize-soybean intercropping is the shading effect of maize, and soybean plants are highly responsive to these shading conditions caused by maize plants (Raza et al., 2020a). Reduced light exposure results in morphological and physiological changes in soybean, such as increased plant height and higher susceptibility to lodging (Li et al., 2014). Lodging of soybean is a major issue in maize-soybean intercropping systems, which inhibits the transportation of photo-assimilates, nutrients and water ultimately, leading to decreased crop yield (Liu et al., 2015; Wu et al., 2017). However, appropriate supply of essential plant nutrients such as iron (Fe), molybdenum (Mo) etc. can alleviate the shading induced adverse impacts on soybean growth, yield and physiology under maize-soybean intercropping. Despite the ongoing researches in improving the shade tolerance, and associated lodging losses in soybean, limited data is available in literature regarding the beneficial impacts of essential plant nutrients such as Mo in improving the shade tolerance in soybean under maize-soybean intercropping system (Oliveira et al., 2022).

Molybdenum (Mo) is an essential micronutrient that serves a crucial role in nitrogen (N) metabolism as a cofactor for nitrate reductase and nitrogenase. (Mendel, 2013; Cakmak et al., 2023). In legumes, Mo is particularly important for N-fixation, as it directly affects nitrogenase functioning in root nodules (Yang et al., 2020). Numerous studies have highlighted the significance of Mo in soybean production (Laltlanmawia et al., 2004; Raut et al., 2004; Kadlag et al., 2006) as well as effects of its combined application with Rhizobium (Sunita et al., 2000; Adkine et al., 2011). Improved crop growth and development in terms of branch number, root nodules, leaf chlorophyll content, grain and straw yield, seed protein, and oil contents have been observed through the use of zinc, iron, and seed priming with Mo (Tiwari et al., 2018). High soybean yields necessitate substantial nitrogen (N) input, with the most cost-effective source of N for soybean being biological nitrogen fixation (BNF) by the symbiotic association between plant and bacteria, mainly belonging to genus *Bradyrhizobium* (Htwe et al., 2019). Interspecific competition between maize-soybean intercropping leads to more efficient utilization of applied N (Zhang et al., 2023). Adding *Bradyrhizobium* with these three micronutrients zinc, iron, and molybdenum have resulted in a remarkable yield response in soybean as reported earlier by Tiwari et al. (2018) and Joglekar et al. (2023). Furthermore, Mo can influence the interaction between maize and soybean in intercropping systems, potentially impacting overall crop productivity and quality (Abendroth et al., 2017; Oliveira et al., 2022). Thus, understanding the role of Mo in maize-soybean intercropping systems is crucial for optimizing crop management practices and maximizing productivity in order to address the existing knowledge gap. Present study aimed at to assess the impact of Mo application on morphological and physiological attributes of soybean intercropped with maize. Hypothesized adequate Mo application and subsequent availability to soybean promotes plant growth, N-fixation, and biomass accumulation, enhancing overall productivity and resource utilization in such systems. These findings are expected to be of practical importance for farmers, agronomists, and researchers in the fields of plant nutrition, crop physiology, and agroecosystems, providing valuable insights to optimize crop management strategies.

## 2 Materials and methods

Current field study was carried out at experimental area of Sindh Agriculture University Tandojam, Hyderabad, Pakistan located at (26.1° N, 68.5° E). Meteorological data of experimental site are presented in **Table 1**. Experimental units were arranged by following randomized complete block design (RCBD) with three replications. Foliar applications of Mo in the form of sodium molybdate (Na_2_MoO_4_) was done at various rates 0, 60, 120 and 180 g ha^-1^), and it was applied during the R1 growth stage of soybean crop by dissolving in 200 L of water.

**Table 1:** Two years month-wise average meteorological (2020–2021) in Tandojam.

Month	Temperature (°C)			Relative Humidity (%)	Sunshine (hour)	Wind Speed (km day^−1^)
	Maximum	Minimum	Mean			
January	24.0	9.10	16.55	68	8.40	21.82
February	27.4	11.4	19.40	64	8.80	18.71
March	33.2	16.0	24.60	58	9.20	21.38
April	38.3	21.2	29.75	54	9.70	35.18
May	40.4	25.4	32.90	61	10.2	50.77
June	44.9	27.2	33.05	67	8.60	64.13
July	46.3	26.9	31.60	74	7.20	52.55
August	38.1	26.2	30.65	77	8.10	32.96
September	35.2	24.4	29.80	75	9.30	48.54
October	35.5	19.8	27.65	69	9.50	13.36
November	31.1	14.9	23.00	64	9.00	16.03
December	25.5	10.7	18.10	67	8.20	18.71

Source: Meteorological station from Agriculture Research Institute Tandojam, Sindh Pakistan.

Physicochemical properties of experimental soils before planting are presented in **Table 2**. Soil texture was determined using Hydrometer method (Bouyoucos, 1962; Qasim et al., 2023). Five grams of soil were mixed with 10 ml 1 N HCl and 50 ml distilled water. Boiled 2 mins, cooled, and 3 drops phenolphthalein added. Titration against 1 N NaOH followed. Calcium carbonate calculated using (Horváth et al., 2005) respectively. Soil EC was determined by soil water extraction (1:2) method by using conductivity meter. Soil pH was measured by soil-water suspension method (U.S. Salinity Laboratory Lab Staff, 1954). Organic carbon was determined by modified Walkley-Black titration and ammonium saturation methods, respectively (Walkley and Black, 1934). Extractable phosphorus was determined using the Olsen method (Olsen and Sommers, 1982). Extractable potassium was determined by mixing 5 g of soil with 1 N ammonium acetate solution, followed by flame photometry analysis (Rhoades, 1983), and available nitrogen was determined by alkaline potassium permanganate method (Wang et al., 2013).

**Table 2:** Physico-chemical properties of experimental soils.

Property	Soil
Texture	Silt clay loam
pH (1:5 soil water extract)	7.42
EC	0.93 dS m^−1^
Calcium carbonate	13.37%
Organic matter	0.85%
Total N	0.07%
Extractable phosphorus	3.62 mg kg^−1^
Extractable Potassium	146.0 mg kg^−1^

### 2.1 Seed procurement and agronomic practices

**Figure 1:**
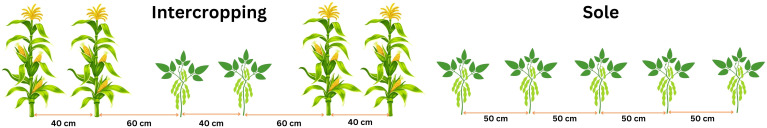
The arrangement of Maize and Soybean crops in strip intercropping and sole cropping systems was varied, with different planting patterns used in each.

Present study utilized two crop varieties, determinate soybean ‘NARC^−1^6’ and semi-compact maize ‘DK-6317’. Seeds were procured from National Agricultural Research Centre (NARC), Pakistan. Two rows of soybean per strip were planted at row-to-row spacing of 40 × 60 cm. Sole soybean plots had row spacing of 50 cm (**Figure 1**). All the agronomic practices were performed manually. Basal fertilization was applied before sowing, with a dose of N (120 kg ha^-1^ urea), P (180 kg ha^-1^ diammonium phosphate), and K (150 kg ha^-1^ sulfate of potash) for maize and N (75 kg ha^-1^ urea), P (150 kg ha^-1^ diammonium phosphate), and K (100 kg ha^-1^ sulfate of potash) for soybean. Two additional N doses were applied at 60 and 100 kg ha^-1^ for maize at the V6 and tasseling stages (Abendroth et al., 2017). Experimental plots were irrigated with 550 ± 100 mm water throughout the experiment using the furrow irrigation method.

### 2.2 Sampling and measurements

#### 2.2.1 Observation of plant morphological characteristics

Root fresh and dry weights were recorded using an analytical balance and were expressed in g plant^−1^. Whereas, stem internodal length, shoot height and root length were measured using a tape. Stem and internode diameter and lodging were also measured using the vernier caliper. Leaf area and number of plants were assessed five times (at 45, 65, 85, 105, and 125 days) after sowing (DAS) in two consecutive years. Five soybean plants were destructively sampled from each plot at each time point. The leaf area was determined by multiplying the maximum width and length of the leaves by a crop-specific coefficient factor of 0.75 for soybean (Gao et al., 2010). Leaf area index (LAI) was then calculated as followed (Raza et al., 2019).


(1) $$LAI=\;\frac{\left(Leaf\;area\;plant^{-1}\times Plant\;number\;plot^{-1}\right)}{Plot\;area}$$

#### 2.2.2 Observation of yield and yield components

Phenological plant stages were observed by collecting five soybean plants from each plot during the study (**Table 3**) that indicated the growth stages of the plants at specific DAS (45, 65, 85, 105, and 125 DAS). Ten soybean plants from each planting pattern were collected for total dry matter accumulation analysis (Raza et al., 2019). Plant parts were separated into root, straw, and grain, and sun-dried for 7-10 days to reach a constant weight. Total dry matter accumulation was determined by summing the dry matter of each part. To measure seed yield, 40 soybean plants were collected and sun-dried, then threshed and weighed to determine yield (kg ha^-1^) (Raven, 1988; Mao et al., 2012).

**Table 3:** Phenological stages observed for soybean in the intercropping system at specific DAS.

DAS	Soybean Growth Stages
45	V2-V3
65	V5
85	R2-R3
105	R5-R6
125	R7

### 2.3 Physiological and biochemical indexes

#### 2.3.1 Photosynthetic indices

Leaf photosynthetic rate (Pn), stomatal conductance (Gs), intercellular CO_2_ concentration (Ci), and transpiration rate (Tr) were measured on sunny days between 09:00-12:00 (local time) from fully expanded top leaves (3 leaves per experimental units from 3 different plants) with a Li-Cor 6400 photosynthesis measuring system. Measurements were performed on the 4^th^ and 5^th^ fully expanded leaves at 100 DAS (R6).

#### 2.3.2 Chlorophyll content analysis

Chlorophyll content was determined during reproductive stages by taking three replication from each leaf of a plant using a portable chlorophyll meter SPAD-502 (Minolta, Japan) (Hong et al., 2005). The value of chlorophyll (Chl) content (mg m^−2^) was estimated from corresponding SPAD values by using the following equation:


(2) $$Chl\;content\;\left(mg\;m^{-2}\right)\;=\;15.68\;\left(SPAD\;unit–\right)\;-\;209.03$$

#### 2.3.3 Antioxidant enzymes

Analyses involving antioxidant enzyme activities were performed in triplicates. Activities of superoxide dismutase (SOD) was measured by using a photochemical reduction of nitro blue tetrazolium (NBT) inhibition method (Kalimuthu et al., 2022), where reaction mixture consisted of a potassium-phosphate buffer (125 mm, pH 7.8), 3 mM MgSO_4_, 3.1 mM EDTA, 2% polyvinyl polypyrrolidone (PVPP), methionine (130 mM), riboflavin (600 µM), NBT (22.5 mM), and plant extract. Reaction was illuminated for 15 min and absorbance was measured at 560 nm. One unit of SOD activity was defined as 50% reduction of A560 control. Catalase (CAT) activity was determined by measuring the decrease in absorbance of reaction mixture containing H_2_O_2_ (100 mM), plant extract, and a potassium-phosphate buffer (125 mm, pH 7.0) at 240 nm (Kalimuthu et al., 2022). Peroxidase (POD) activity was determined by measuring the increase in absorbance of reaction mixture consisting of ascorbate solution (5 mM), H_2_O_2_ (100 mM), EDTA solution (1 mM), plant extract, and a potassium-phosphate buffer (125 mm, pH 7.0) at 290 nm (Probst et al., 2021). In addition, malondialdehyde contents (MDA) in soybean leaves were determined by following the method of Sheng and Zhu (2019), which involved the homogenization of soybean leaves in the presence of trichloroacetic acid (TCA; 10%) followed by their centrifugation at 9000*g* for 20 minutes. Reaction mixture for this assay comprised of 2 mL of aliquot, 2 mL of 0.6% thiobarbituric acid, and heated for 20 minutes at 100°C followed by their abrupt cooling in an ice bath. Finally, absorbance of the mixture was checked at 532 and 450 nm for MDA determination.

### 2.4 Determination of N, P, K, and Mo contents in different parts of the plant

At physiological maturity, 30 soybean and 15 maize plants were randomly selected from each experimental unit, and were separated into grain, straw and roots, and dried at 70°C for 72 h to determine N, P, K, and Mo content. The N concentration was determined using the Kjeldahl Apparatus, P concentration was estimated using the Vanadomolybdate method (Xia et al., 2013), and K concentration was measured using the FAAS method (Varian 250 plus). The NPK uptake was calculated by multiplying the biomass of each organ by the N, P, and K concentrations, and presented as kilogram per hectare (Mao et al., 2012). Similarly, for Mo analysis, approximately 0.2 g of dried tissue was microwave-digested in 5 mL HNO_3_ (4/1 *v*/*v*) at 160°C for 45 min and diluted, and Mo content was quantified using inductively coupled plasma optical emission spectroscopy (ICP-OES) (Perkin Elmer Optima 8300, Massachusetts, USA).

### 2.5 Statistical analysis

Data was analyzed for normal distribution and homogeneity of variance. Log transformations were applied where necessary to correct deviations from these assumptions. Analysis was performed using Microsoft Excel 2013 (Microsoft Corp. Washington, USA), IBM SPSS Statistics V22.0 (SPSS Inc., Chicago, New Mexico, USA). One-way analysis of variance (ANOVA) was used to statistically analyze the obtained data. Later, data was arranged in MS Excel 2013, and their relevant means and standard errors were also computed by MS Excel 2013.

## 3 Results

### 3.1 Growth and yield attributes

**Figure 2:**
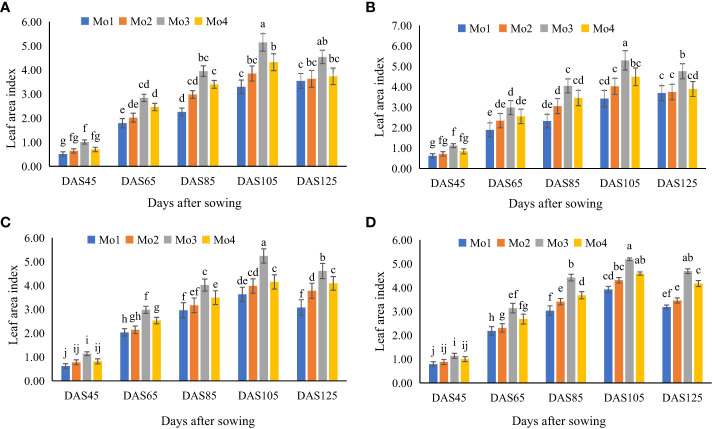
Effect of different levels of Mo on leaf area index (m^2^) of soybean at different time intervals, **(A)** sole soybean during 2020, **(B)** sole soybean during 2021, **(C)** soybean intercropped during 2020 and **(D)** soybean intercropped during 2021. The columns sharing same letters are statistically non-significant (Tukey’s HSD test) at *p*< 0.05. The error bars indicate standard deviation.

**Figure 3:**
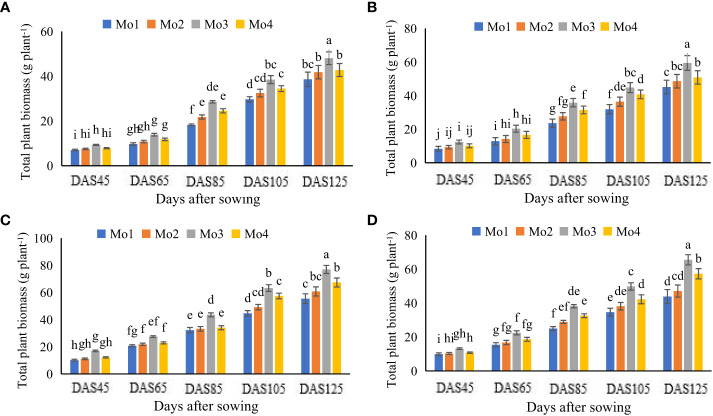
Effect of different levels of Mo on total plant biomass (g) at different time intervals, **(A)** sole soybean during 2020, **(B)** sole soybean during 2021, **(C)** soybean intercropped during 2020 and **(D)** soybean intercropped during 2021. The columns sharing same letters are statistically non-significant (Tukey’s HSD test) at *p*< 0.05. Error bars indicate standard deviation.

Results regarding growth attributes revealed a significant impact of Mo application in both years under both cropping systems (**Figures 2**, **3**). However, Mo application @ 120 g ha^-1^ proved to be the most significant in this regard. It was observed that in sole soybean plantation, maximum increment in growth attributes such as leaf area index (LAI; 436 and 393%), plant height (16 and 12%), root length (107 and 102%), nodules per plant (18 and 17%), stem diameter (15 and 16%), total plant biomass (TPB; 454 and 364%), yield (19 and 18%) were observed as compared to control treatment after 105 DAS during year 2020 and 2021. Similarly, in soybean-maize intercropping system, Mo application @ 120 g ha^-1^ enhanced the LAI (434 and 441%), TPB (430 and 461%), plant height (12 and 13%), root length (94 and 86%), nodules per plant (14 and 15%), stem diameter (12 and 14%), and yield (15 and 20%) after 125 DAS during year 2020 and 2021, respectively (**Tables 4**, **5**). On average, more improvement in all the plant growth and yield attributes was evident after Mo application during 2021 as compared to the preceding year (2020).

**Table 4:** Soybean yield in maize-soybean intercropping system.

Treatments	2020		2021	
	Sole Soybean	Soybean Intercropped	Sole Soybean	Soybean Intercropped
Mo1	906.7 c	521.6 c	1014.8 d	631.1 c
Mo2	1008.2 bc	558.5 bc	1079.7 c	653.1 bc
Mo3	1122.4 a	626.0 a	1192.9 a	725.3 a
Mo4	1065.1 ab	592.4 ab	1136.2 b	688.6 b
P value (0.05)	0	0	0.0001	0

Different lowercase letters, in a single column, show significant differences among concentration means at p< 0.05.

**Table 5:** Morphological properties of soybean under sole and intercropping with maize in different (plant^−1^) during 2020 and 2021.

Sole Soybean										
2020						2021				
T	PH	RL	NoP	SDIM	IL	PH	RL	NoP	SDIM	IL
Mo1	55.5 ± 0.81 d	81.4 ± 2.18 d	23.9 ± 0.46 d	5.3 ± 0.22 c	5.4 ± 0.10 b	57.6 ± 1.71 c	88.2 ± 1.32 d	24.7 ± 0.42 d	5.5 ± 0.14 c	5.6 ± 0.21 b
Mo2	58.4 ± 0.83 c	104.3 ± 1.36 c	25.3 ± 0.57 c	5.6 ± 0.28 bc	5.3 ± 0.09 ab	60.7 ± 1.98 b	112.4 ± 1.41 c	26.2 ± 0.52 c	5.8 ± 0.19 bc	5.5 ± 0.26 ab
Mo3	64.4 ± 0.94 a	168.5 ± 2.35 a	27.9 ± 0.78 a	6.1 ± 0.41 a	5.0 ± 0.12 a	65.9 ± 1.14 a	178.3 ± 8.23 a	29.0 ± 0.71 a	6.4 ± 0.33 a	5.2 ± 0.34 a
Mo4	61.6 ± 1.19 b	136.2 ± 1.14 b	26.7 ± 0.66 b	5.9 ± 0.29 ab	5.1 ± 0.07 ab	65.1 ± 1.65 a	139.7 ± 1.22 b	27.7 ± 0.62 b	6.1 ± 0.20 ab	5.3 ± 0.30 a
P value (0.05)	0	0.0345	0	0.0037	0.1015	0.0001	0.0135	0	0.0024	0.0476
Soybean intercropping										
Mo1	77.1 ± 2.20 d	96.2 ± 1.17	34.7 ± 1.03 d	7.4 ± 0.49 c	4.1 ± 0.18 d	80.3 ± 2.17 d	105.03 ± 2.34 d	36.0 ± 0.61 d	7.6 ± 0.43 c	4.3 ± 0.23 c
Mo2	80.9 ± 2.32 c	129 ± 1.62	36.4 ± 1.09 c	7.8 ± 0.39 bc	4.2 ± 0.14 c	84.3 ± 2.29 c	138.23 ± 1.42 c	37.8 ± 0.64 c	8.0 ± 0.31 bc	4.4 ± 0.28 b
Mo3	86.6 ± 2.66 a	186.2 ± 1.24	39.5 ± 1.15 a	8.3 ± 0.34 a	4.5 ± 0.16 a	90.4 ± 2.13 a	195.11 ± 2.32 a	41.2 ± 0.70 a	8.7 ± 0.25 a	4.6 ± 0.29 a
Mo4	83.8 ± 3.03 b	155.6 ± 1.01	38.1 ± 1.15 b	8.1 ± 0.53 ab	4.4 ± 0.14 b	87.4 ± 1.44 b	164.45 ± 1.41 b	39.7 ± 0.68 b	8.4 ± 0.46 ab	4.5 ± 0.30 ab
P value (0.05)	0	0.0125	0	0.015	0	0.0001	0.015	0	0.012	0.0015

T, Treatments; PH, Plant height; RL, Root length; NoP, number of nodules plant^−1^; SDIM, steam diameter (mm); IL, internode length (inch). Different lowercase letters, in a single column, show significant differences among concentration means at p< 0.05.

### 3.2 Physiological attributes

**Figure 4:**
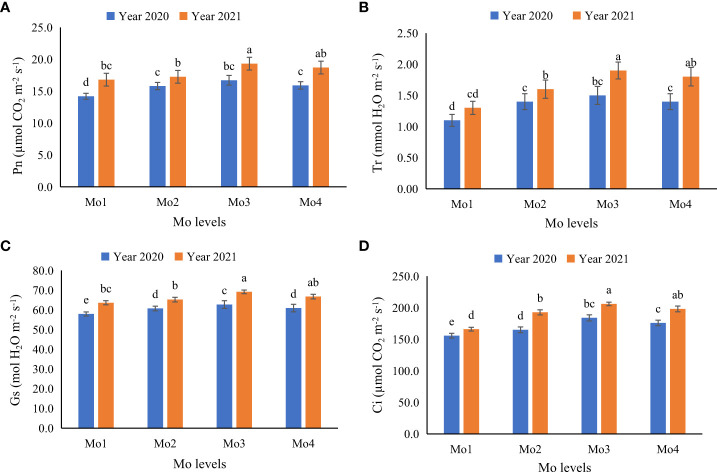
Effect of different levels of Mo on gaseous exchange attributes of soybean leaves at different Mo application levels for sole spybean, **(A)** Photosynthetic rate (Pn; µmol CO_2_ m^−2^ s^−1^), **(B)** Transpiration rate (Tr; mmol H_2_O m^−2^ s^−1^), **(C)** Stomatal conductance (Gs; mol H_2_O m^−2^ s^−1^) and **(D)** inter-cellular CO_2_ concentration (Ci; µmol CO_2_ m^−2^ s^−1^). The columns sharing same letters are statistically non-significant (Tukey’s HSD test) at *p*< 0.05. Error bars indicate standard deviation.

**Figure 5:**
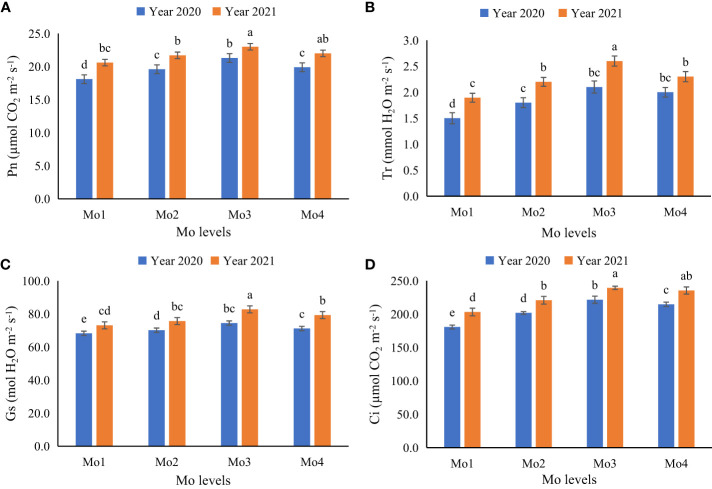
Effect of different levels of Mo on gaseous exchange attributes of soybean leaves at different Mo application levels for intercropping soybean, **(A)** Photosynthetic rate (Pn; µmol CO_2_ m^−2^ s^−1^), **(B)** Transpiration rate (Tr; mmol H_2_O m^−2^ s^−1^), **(C)** Stomatal conductance (Gs; mol H_2_O m^−2^ s^−1^) and **(D)** inter-cellular CO_2_ concentration (Ci; µmol CO_2_ m^−2^ s^−1^). The columns sharing same letters are statistically non-significant (Tukey’s HSD test) at *p*< 0.05. Error bars indicate standard deviation.

**Figure 6:**
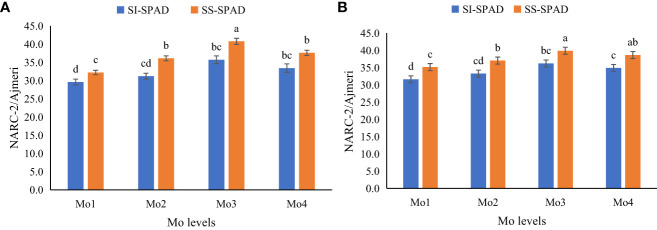
Effect of different levels of Mo on SPAD value of soybean leaves grown solely and intercropped with maize, **(A)** SPAD value in 2020 and **(B)** SPAD value in 2021. The columns sharing different letters are statistically non-significant (Tukey’s HSD test) at *p*< 0.05. Error bars indicate standard deviation. SI, Soybean intercropped; SS, Sole Soybean.

In terms of plant physiological attributes, it was observed that exogenous application of Mo led to a significant improvement in all the measured physiological attributes i.e., transpiration rate, stomatal conductance, inter-cellular CO_2_ concentration, and photosynthetic rate, as well as chlorophyll content (SPAD value). However, application of Mo @ 120 g ha^-1^ yielded significantly better results as compared to other Mo levels. It was observed that during year 2020, under sole soybean plantation and soybean-maize intercropping, application of Mo @ 120 g ha^-1^ improved transpiration rate (12 and 15%), stomatal conductance (9 and 10%), inter-cellular CO_2_ concentration (13 and 11%), and photosynthetic rate (11 and 11%) respectively. However, during 2021, more promising outcomes were pragmatic in terms of physiological attributes i.e., transpiration rate, stomatal conductance, inter-cellular CO_2_ concentration, and photosynthetic rate. Under both sole and intercropped soybean, application of Mo @ 120 g ha^-1^ significantly enhanced the physiological attributes i.e., transpiration rate (18 and 19%), stomatal conductance (11 and 11%), inter-cellular CO_2_ concentration (13 and 12%), and photosynthetic rate (12 and 11%) respectively as compared to control treatment (**Figures 4**–**6**). It was obvious from the results of physiological attributes that more promising outcomes of Mo application @ 120 g ha^-1^ were observed under soybean-maize intercropping during both the years.

### 3.3 Antioxidant activities

**Figure 7:**
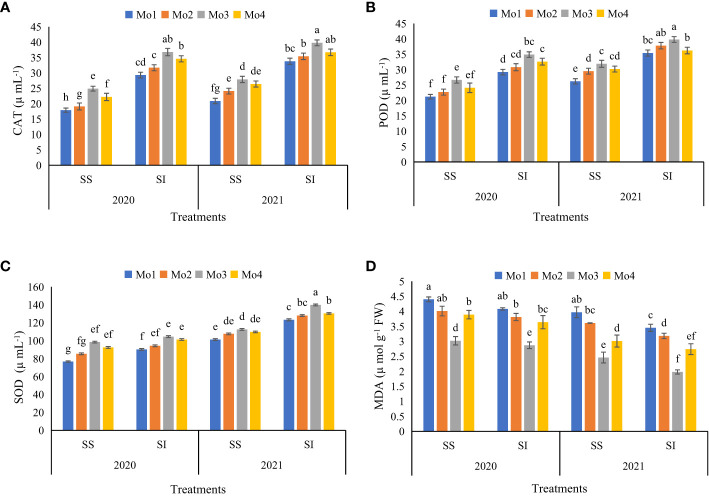
Effect of different levels of Mo on antioxidant activities **(A)** Catalase, **(B)** Peroxidase, **(C)** Superoxide dismutase and **(D)** malondialdehyde contents in soybean leaves during year 2020–2021. The columns sharing different letters are statistically non-significant (Tukey’s HSD test) at *p*< 0.05. Error bars indicate standard deviation.

Application of Mo also showed significantly positive effects on various antioxidant activities in soybean leaves such as catalase (CAT), peroxidase (POD), and superoxide dismutase (SOD) during sole and intercropping with maize over two consecutive years (2020–2021). During sole plantation in 2020-21, Mo application at a rate of 120 g ha^-1^ led to significant increment in antioxidant enzyme activities CAT (14 and 15%), SOD (13 and 12%), and POD (7 and 5%) respectively, as compared to control treatment. However, in soybean maize intercropping, Mo application @ 120 g ha^-1^ yielded more significant outcomes in all the measured antioxidant activities, including CAT (11 and 12%), POD (5 and 6%), and SOD (11 and 11%) as compared to control treatment (**Figure 7**). Furthermore, lipid oxidation was evaluated in terms of malondialdehyde (MDA) contents in soybean leaves. Results revealed that Mo application at a rate of 120 g ha^-1^ significantly reduced MDA contents in soybean leaves, indicating a significant decrease in lipid peroxidation. In the sole and intercropped soybean cropping systems, Mo application at a rate of 120 g ha ^-1^ during 2020 led to significant reduction in MDA contents by 30 and 31%, respectively as compared to control treatment. However, during the subsequent year (2021), MDA contents were further decreased by 38 and 43% respectively, in the sole and intercropped soybean cropping systems with the application of Mo @ 120 g ha^-1^ (**Figure 7**).

### 3.4 Nutritional attributes

Results in terms of nutritional attributes of soybean such as nitrogen (N), phosphorous (P), potassium (K), and Mo contents were significantly affected by Mo treatments. However, among different application rates, Mo application @ 120 g ha^−1^ was found to be the most efficient in both cropping systems. During year 2020, Mo application at 120 g ha^-1^ significantly increased the N, P, K and Mo contents in roots (10, 13, 13 and 15%), straw (7, 13, 10 and 17%), and grains (10, 10, 10 and 20%) of soybean grown solely (**Table 6**). Whereas, in the same year during intercropping with maize, NPK and Mo contents in roots (13, 13, 17 and 16%), straw (9, 10, 17 and 16%) and grains (11, 12, 26 and 18%) of soybean were also significantly increased under Mo application rate of 120 g ha^-1^ (**Table 6**). Furthermore, during year 2021, relatively more significant outcomes in terms of nutrient uptake were pragmatic, where nutrient uptake in different parts of sole and intercropped soybean were significantly increased after the application of Mo @ 120 g ha^-1^ i.e., NPK and Mo contents in roots (15, 13, 12 and 15% as well as 19, 17, 24 and 17%), straw (12, 13, 14 and 18% as well as 17, 12, 18 and 17%) and grains (12, 14, 14 and 21% as well as 15, 14, 18 and 19%) were increased respectively in sole grown and intercropped soybean crop as compared to control treatment during year 2021 (**Table 6**).

**Table 6:** Total nitrogen, phosphorus, potassium, and molybdenum uptake under soybean grown as sole crop in different parts of the soybean (plant^−1^) during 2020 and 2021.

		2020			2021		
Nutrients	Treatments	Root	Straw	Grain	Root	Straw	Grain
Nitrogen	Mo1	0.31 ± 0.03 b	0.48 ± 0.03 b	0.85 ± 0.05 bc	0.34 ± 0.03 c	0.58 ± 0.04 c	0.89 ± 0.04 c
	Mo2	0.35 ± 0.03 ab	0.51 ± 0.03 b	0.88 ± 0.06 b	0.40 ± 0.03 b	0.62 ± 0.04 b	0.93 ± 0.06 b
	Mo3	0.41 ± 0.02 a	0.62 ± 0.03 a	0.96 ± 0.05 a	0.47 ± 0.03 a	0.68 ± 0.03 a	1.01 ± 0.05 a
	Mo4	0.38 ± 0.03 ab	0.57 ± 0.03 ab	0.91 ± 0.05 ab	0.42 ± 0.03 ab	0.65 ± 0.03 ab	0.97 ± 0.05 ab
P value (0.05)		0.009	0.0025	0.0017	0.0055	0.0025	0.0006
Phosphorus	Mo1	0.03062 ± 0.00 b	0.0447 ± 0.00 c	0.0967 ± 0.02 bc	0.0322 ± 0.00 b	0.0560 ± 0.00 b	0.1443 ± 0.02 c
	Mo2	0.03412 ± 0.00 ab	0.0561 ± 0.00 b	0.1112 ± 0.02 b	0.0369 ± 0.00 ab	0.0589 ± 0.00 b	0.1504 ± 0.02 b
	Mo3	0.03811 ± 0.00 a	0.0618 ± 0.00 a	0.1521 ± 0.02 a	0.0399 ± 0.00 a	0.0632 ± 0.00 a	0.1633 ± 0.02 a
	Mo4	0.03510 ± 0.00 ab	0.0582 ± 0.00 ab	0.1361 ± 0.02 ab	0.0384 ± 0.00 ab	0.0611 ± 0.00 ab	0.1568 ± 0.02 ab
P value (0.05)		0.4547	0.0701	0.0079	0.4547	0.0701	0.0029
Potassium	Mo1	0.0420 ± 0.00 ab	0.5441 ± 0.07 b	0.2891 ± 0.02 b	0.0438 ± 0.00 b	0.5558 ± 0.06 c	0.2997 ± 0.01 c
	Mo2	0.0432 ± 0.00 ab	0.5585 ± 0.07 ab	0.2969 ± 0.02 b	0.0459 ± 0.00 ab	0.5865 ± 0.07 b	0.3156 ± 0.02 b
	Mo3	0.0479 ± 0.00 a	0.5994 ± 0.07 a	0.3177 ± 0.02 a	0.0492 ± 0.00 a	0.6362 ± 0.08 a	0.3419 ± 0.03 a
	Mo4	0.0450 ± 0.00 ab	0.5753 ± 0.07 ab	0.3054 ± 0.02 ab	0.0477 ± 0.00 ab	0.6112 ± 0.08 ab	0.3292 ± 0.02 ab
P value (0.05)		0.4547	0.0003	0.0219	0	0.0049	0.0219
Molybdenum	Mo1	16.40 ± 0.26 d	22.47 ± 0.18 d	46.84 ± 0.36 d	18.09 ± 0.27 d	23.30 ± 0.13 d	48.96 ± 0.49 d
	Mo2	17.35 ± 0.20 c	23.14 ± 0.18 c	49.93 ± 0.40 c	19.14 ± 0.22 c	24.69 ± 0.10 c	52.36 ± 0.55 c
	Mo3	20.62 ± 0.03 a	26.19 ± 0.24 a	56.19 ± 0.43 a	22.16 ± 0.04 a	28.41 ± 0.14 a	60.10 ± 0.61 a
	Mo4	19.32 ± 0.14 b	24.06 ± 0.18 b	53.22 ± 0.42 b	20.28 ± 0.15 b	26.17 ± 0.10 b	55.92 ± 0.58 b
P value (0.05)		0	0	0	0	0	0

Different lowercase letters, in a single column, show significant differences among concentration means at p< 0.05.

## 4 Discussion

Effect of Mo application on soybean growth and yield characteristics has been studied extensively in recent years (Bittner, 2014; Qin et al., 2017; Mahilane and Singh, 2018; Quddus et al., 2020; Rana et al., 2020; Ali et al., 2021; Oliveira et al., 2022; Nasar et al., 2022a). In present study, application of Mo at different levels were tested in sole and intercropping with maize. Observations revealed a positive correlation between Mo application and LAI, consistent with the findings of (Nasar et al., 2022a; Oliveira et al., 2022), and may be attributed to Mo mediated improvement of photosynthetic efficiency (Gao et al., 2010). Increased total plant biomass as observed in current study is also consistent with the findings of previous researchers (Raza et al., 2020b; Hussain et al., 2021), which might be attributed to the role of Mo in N metabolism, enhancing N-use efficiency and promoting plant growth (Probst et al., 2021). Additionally, higher biomass values in intercropping compared to sole cropping systems may be due to the efficient utilization of resources, such as light, water, and nutrients as suggested by (Gao et al., 2010). Similarly, positive correlation between Mo application and various morphological plant attributes are in agreement with the previously reported studies (Qin et al., 2017; Heshmat et al., 2021; Nasar et al., 2022a), which might be correlated with the enhanced application and subsequent role of Mo in various enzymatic processes related to plant growth and development (Bittner, 2014; Probst et al., 2021). Moreover, improvement in these traits in intercropping system suggests that Mo application can enhance the compatibility of soybean in intercropping systems, ensuring better growth and yield (Ren et al., 2017).

In current study, Mo application also improved soybean yield in both sole and intercropping system, which is also consistent with the outcomes of previous researchers (Qin et al., 2017; Mahilane and Singh, 2018; Rana et al., 2020; Ali et al., 2021; Nasar et al., 2022b). However, lower yields were observed during intercropping than in sole soybean cultivation, which might be related to provision of competition between maize and soybean for resources (Ren et al., 2017; Raza et al., 2020b). However, increased grain yield with increasing Mo levels in intercropping treatments suggests that Mo can mitigate the adverse effects of intercropping on soybean yield (Oliveira et al., 2022). Additionally, higher aggregate outputs observed in intercropping treatments with higher Mo levels align with previous studies, indicating the potential for increased yield in intercropping systems (Ren et al., 2017; Raza et al., 2019; Raza et al., 2020b). In addition to that, improved soybean physiology after Mo application in both years under sole and intercropping systems was due to the role of Mo as an essential component of the enzyme nitrogenase which is involved in N-fixation (Zuber and Kang, 1978), which contributed to the improvement in the efficiency of photosynthetic apparatus as well as stomatal conductance (Wu et al., 2017). Similar results were previously reported by (Kaiser et al., 2005; Kadlag et al., 2006). Furthermore, increase in chlorophyll content as observed in this study could be linked to the role of Mo in the formation of Mo-cofactor, which is involved in the synthesis of chlorophyll and other essential biomolecules (Mendel, 2013; Probst et al., 2021), as suggested by Li et al. (2018). Moreover, positive influence of Mo on antioxidant activities in soybean was also pragmatic in current study, which has been earlier reported by (Tiwari et al., 2018; Oliveira et al., 2022). In contrast, while the application of Mo led to a decline in lipid peroxidation through potential enhancement of antioxidant enzyme activities in soybean plants, facilitating ROS scavenging and protection against oxidative stress, plants can also mobilize intrinsic defense mechanisms to counteract ROS, including the activation of antioxidant enzymes and the synthesis of osmotic adjustment compounds, as highlighted by (Lv et al., 2019; Jia et al., 2021; Wang et al., 2023) in their respective studies.

Positive interaction between Mo application and nutritional uptake by plants was observed, as previously reported by (Raza et al., 2019). Mo is an essential micronutrient for plants and plays a crucial role in N metabolism (Walkley and Black, 1934). It has been reported that Mo application enhances N accumulation, seed yield, and seed protein content in soybean (Campo et al., 2009) as was observed in current study. Previous research are supported by our results, which show an increase in plant nutrient profile quality under both the solo and intercropping systems. (Raut et al., 2004; Weisany et al., 2015; Zhao et al., 2022). Moreover, Mo has been reported to exert positive effects on photosynthesis (Probst et al., 2021), which may further contribute to enhanced nutrient uptake as observed in this study. These improvements in the soybean growth, yield, morphology, physiology, and antioxidant activities after Mo application have significant implications for agricultural productivity, particularly in maize-soybean intercropping systems. Improved photosynthetic performance and stomatal conductance can contribute to increased biomass and grain yields (Wolff and Coltman, 1990; Raza et al., 2021; Mirriam et al., 2022). Moreover, enhanced antioxidant activities can help improve the stress tolerance of soybean plants under the shaded conditions commonly encountered in intercropping systems (Zuber and Kang, 1978; Li et al., 2014; Wu et al., 2017). As a result, Mo application can potentially enhance the overall productivity and sustainability of maize-soybean intercropping systems (Gao et al., 2010; Liu et al., 2015; Raza et al., 2020b).

## 5 Conclusions

Intercropping of soybean and maize has numerous benefits but shading stress on soybean due to maize canopy can negatively impact its growth and yield. Exogenous application of Mo has shown considerable potential in mitigating the negative effects of shading stress and improving soybean growth and yield. Molybdenum (Mo) is directly involved in photosynthetic process of plants and it also stimulates the assimilatory enzyme activities under stressed conditions, and in this way, help the plant to sustain shading stress. In a field trial, different levels of Mo were applied and evaluated for their effects on growth and yield parameters. Results showed that all the tested levels of Mo significantly improved soybean growth and yield, with the most efficient level being 120 g ha^-1^. These findings have significant implications for the development of sustainable intercropping practices in agriculture, which can promote optimal land utilization and enhance crop productivity, contributing to food security and environmental sustainability. Application of Mo proved to be a suitable alternative in improving the shade tolerance in soybean under soybean maize intercropping system. Following schematic diagram shows the mechanism of induced shade tolerance in soybean after Mo application.

Soybean-maize intercropping
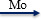
improved photosynthesis and enzyme activities
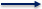
shade tolerance
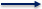
improved plant growth and physiology.

## Data availability statement

The original contributions presented in the study are included in the article/supplementary material. Further inquiries can be directed to the corresponding author.

## Author contributions

Conceptualization, ZJ and SC. Methodology, SA. Software, MQ. Validation, ZJ, SA and MQ. Formal analysis, SA. Investigation, ZJ. Resources, SA. Data curation, MA. Writing—original draft preparation, ZJ. Writing—review and editing, MQ, JD and YW. Visualization, SA. Supervision, SC. Project administration, SC. Funding acquisition, SC. All authors have read and agreed to the published version of the manuscript.
